# IL-6/IL-12 Cytokine Receptor Shuffling of Extra- and Intracellular Domains Reveals Canonical STAT Activation via Synthetic IL-35 and IL-39 Signaling

**DOI:** 10.1038/s41598-017-15173-3

**Published:** 2017-11-09

**Authors:** D. M. Floss, M. Schönberg, M. Franke, F. C. Horstmeier, E. Engelowski, A. Schneider, E. M. Rosenfeldt, J. Scheller

**Affiliations:** Institute of Biochemistry and Molecular Biology II, Medical Faculty, Heinrich-Heine-University, 40225 Düsseldorf, Germany

## Abstract

IL-35 and IL-39 are recently discovered shared members of the IL-6- and IL-12–type cytokine family with immune-suppressive capacity. IL-35 has been reported to induce the formation of four different receptor complexes: gp130:IL-12β2, gp130:gp130, IL-12β2:IL-12β2, and IL-12β2:WSX-1. IL-39 was proposed to form a gp130:IL-23R receptor complex. IL-35, but not IL-39, has been reported to activate non-conventional STAT signaling, depending on the receptor complex and target cell. Analyses of IL-35 and IL-39 are, however, hampered by the lack of biologically active recombinant IL-35 and IL-39 proteins. Therefore, we engineered chimeric cytokine receptors to accomplish synthetic IL-35 and IL- 39 signaling by shuffling the extra- and intracellular domains of IL-6/IL-12–type cytokine receptors, resulting in biological activity for all previously described IL-35 receptor complexes. Moreover, we found that the proposed IL-39 receptor complex is biologically active and discovered two additional biologically active synthetic receptor combinations, gp130/IL-12Rβ1 and IL-23R/IL-12Rβ2. Surprisingly, synthetic IL-35 activation led to more canonical STAT signaling of all receptor complexes. In summary, our receptor shuffling approach highlights an interchangeable, modular domain structure among IL-6- and IL-12–type cytokine receptors and enabled synthetic IL-35 and IL-39 signaling.

## Introduction

**Figure 1:**
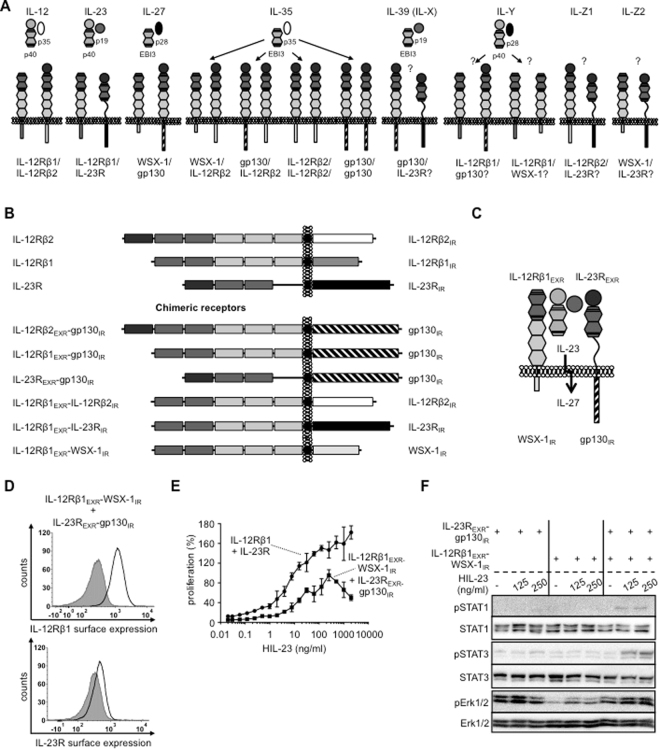
Chimeric receptors of the IL-12/IL-6 cytokine family. (**A**) Approved and potential receptor combinations of the IL-12/IL-6 cytokine family. Schematic overview of all possible IL-12Rβ1/IL-12Rβ2/IL-23R/WSX-1/gp130 receptor combinations with the respective stimulatory cytokine tested in this study. (**B**) Schematic overview of murine IL-12Rβ1/IL-12Rβ2/IL-23R and the chimeric variants with extracellular domains (EXR) of IL-12Rβ1/IL-12Rβ2/IL-23R and intracellular regions (IR) of IL-12Rβ2/IL-23R/gp130/WSX-1. (**C**) Schematic overview of IL-27-type signaling by IL-23-induced receptor activation of IL-12Rβ1_EXR_-WSX-1_IR_ and IL-23R_EXR_-gp130_IR_. (**D**) Representative histograms of IL-12Rβ1_EXR_-WSX-1_IR_ (upper panel) and IL-23R_EXR_-gp130_IR_ (lower panel) surface expression of Ba/F3-gp130/IL-12Rβ1_EXR_-WSX-1_IR_/IL-23R_EXR_-gp130_IR_ cells (light solid lines). Gray-shaded areas indicate Ba/F3-gp130 cells (negative control). (**E**) Cellular proliferation of Ba/F3-gp130/IL-12Rβ1_EXR_-WSX-1_IR_/IL-23R_EXR_-gp130_IR_ and Ba/F3-gp130/IL-12Rβ1/IL-23R cells. Equal numbers of cells were cultured for 3 days in the presence of HIL-23 (0.01 to 2000 ng/ml). Proliferation was measured using the colorimetric CellTiter-Blue Cell Viability Assay. HIL-6–induced proliferation (10 ng/ml) was set to 100%. One representative experiment out of four is shown. Error bars represent SD. (**F**) Analysis of STAT1/3 and Erk1/2 activation. Ba/F3-gp130/IL-12Rβ1_EXR_-WSX-1_IR_, Ba/F3-gp130/IL-23R_EXR_-gp130_IR_ and Ba/F3-gp130/IL-12Rβ1_EXR_-WSX-1_IR_/IL-23R_EXR_-gp130_IR_ cells were washed three times, starved, and stimulated with 125 and 250 ng/ml HIL-23 for 30 min. Cellular lysates were prepared, and equal amounts of total protein (50 μg/lane) were loaded on SDS gels, followed by immunoblotting using specific antibodies for phospho-STAT1/3/Erk1/2 and STAT1/3/Erk1/2. Western blot data show one representative experiment out of two.

The IL-12 family of cytokines is comprised of IL-12, IL-23, IL-27, IL-35 and IL-39 and belongs to the type 1 family of hematopoietic cytokines. IL-27, IL-35 and IL-39 are also designated to the IL-6-type cytokine family^1^. IL-12-type cytokines consist of soluble heterodimers. The cytokine α chains IL-23_p19, IL-12_p35 and IL-27_p28 are structurally related to IL-6 and form complexes with the soluble receptor subunits p40 and EBI3 (β chains). The subunits of IL-12 (p35:p40) and IL-23 (p19:p40) are connected by a disulfide bridge with IL-12_p35 or IL-23_p19 and p40, respectively. The α chain IL-27_p28 and EBI3 form IL-27 without disulfide connection and represents a shared cytokine of the IL-6- and IL-12-type cytokine family, because it signals via the IL-6-type cytokine receptors gp130 and WSX-1^2^. Interaction of IL-12_p35 and EBI3 has been shown in 1997^3^, however, functional rediscovery and naming to IL-35 occurred 10 years later^4^. For IL-35, IL-12_p35 and EBI3 were not connected by a disulfide bridge^5^. IL-39 (also named IL-X) is composed of IL-23_p19 and EBI3, which might be linked by a disulfide bridge^6^. Recently, a synthetic member of the IL-12 cytokine family was generated as single chain molecule and termed IL-Y (IL-27_p28 + p40)^7,8^. Whereas IL-12 and IL-23 signal via the common IL-12-type family receptor complexes IL-12Rβ1:IL-12Rβ2 and IL-12Rβ1:IL-23R, respectively, IL-35 engages receptors from both the IL-6- and IL-12-type family. The receptor complexes of IL-39 (IL-X) and IL-Y are not known, however, combinations of IL-23R:gp130 and WSX-1:IL-12Rβ1, respectively, were recently proposed^9^. However, binding of IL-Y might also induce complex formation of gp130:IL-12Rβ1^10^ (Fig. 1A).

Most cytokines have a unique, high affinity receptor signaling complex and at least in some cases a second lower affinity receptor complex^1^. Interestingly, IL-35 is different, because four receptor complexes have been described: IL-12Rβ2:gp130, IL-12Rβ2:IL-12Rβ2, gp130:gp130 and IL-12Rβ1:WSX-1. Even though studies described the biological function of IL-35 *in vivo* and discovered the aforementioned IL-35 receptor complexes^11–13^, detailed *in vitro* analysis of cytokine-receptor binding and signal transduction analysis are still missing.

Typically, cytokines have defined binding sites and in many cases, single amino acid exchanges reduce or completely disturb cytokine:cytokine-receptor interaction^14^. This does not account for IL-35, because the binding of IL-12_p35 and EBI3 could not be interrupted by introduction of class-typical point mutations^5^. Therefore, binding of IL-12_p35 to EBI3 remains mysterious. Remarkably, in this study IL-35 was analyzed in cell lysates rather than in cell culture supernatants, mainly because IL-35 was very poorly if at all secreted^5^. Our own studies also failed to detect IL-35 (as single components and as Hyper-cytokine fusion protein) in cell culture supernatants and we were not able to stimulate Ba/F3 cells expressing IL-12Rβ2, WSX-1 and gp130 with purified, reconstituted recombinant IL-35^15^. Thus far, only one group succeeded to express and purify tiny amounts of recombinant IL-35 in insect cells, which was biologically active on murine primary T and B cells. Due to formation of p35:p35 and EBI3:EBI3 homodimers, the overall efficacy of p35:EBI3 heterodimer formation was very low^13^. Also commercially available IL-35 failed to induce proliferation of Ba/F3-cells expressing WSX-1, gp130 and IL-12Rβ2 (Fig. S1). After all, protocols to express and purify IL-35 and also IL-39 are still missing.

IL-12, IL-23, IL-27 and IL-39 have family-typical STAT activation patterns, whereas STAT activation of IL-35 diverged^6,16^. IL-12 activates mainly STAT4, but also STAT1, 3 and 5 and IL-23 activates mainly STAT3 but also STAT1, 4 and 5, whereas IL-27 induces STAT1, 3, 5 but not STAT4 and IL-6 activates STAT1, 3 and also STAT5 albeit to a lesser extend^16^. IL-39 induced mainly STAT1 and STAT3^6^. Paradoxically, even though IL-35 share the receptor chains with IL-12, IL-6 and IL-27, IL-35 activates only STAT1 and STAT4, when signaling through a gp130/IL-12Rβ2 heterodimer, and solely STAT1 via a gp130 homodimer or STAT4 via an IL-12Rβ2 homodimer^11^. Moreover, signaling via an IL-12Rβ2/WSX-1 heterodimer induced STAT1, 3, 4 and 5 in T cells and STAT1, 3, and 5 in B cells^13^. It is, however, completely unknown how the different IL-35 induced STAT activation pattern are executed and regulated on the receptor/cellular level.

Due to these limitations, we developed an alternative strategy to analyze IL-35 signal transduction in the well-established, pre-murine B-cell-line Ba/F3, which was commonly used to investigate the signal transduction of cytokines of the IL-6/IL-12 family^17,18^. To this end we engineered shuffled IL-6/IL-12 type cytokine receptors that were responsive to extracellular IL-12/IL-23 stimulation and induced intracellular IL-35/IL-39-signal transduction. We refer to this strategy as synthetic signaling. Using this approach, we were able to confirm biological activity of all described IL-35- and the proposed IL-39-receptor complexes in Ba/F3 cells. Furthermore, we identified two additional receptor combinations of the IL-6/IL-12-type cytokine receptors.

## Results

### Generation, expression and cell surface localization of synthetic IL-12 type receptor chimeras

Cytokines of the IL-12 family signal via five different receptors and eight different receptor complexes, namely IL-12Rβ1:IL-12Rβ2 for IL-12, IL-23R:IL-12Rβ1 for IL-23, WSX-1:gp130 for IL-27 and IL-12Rβ2:gp130, IL-12Rβ2:IL-12Rβ2, gp130:gp130 and IL-12Rβ2:WSX-1 for IL-35, proposed IL-23R:gp130 for IL-39 (Fig. 1A). Typical analysis of signal transduction pathways by IL-35- and IL-39-stimulation of established cell lines with defined expression of the different receptor combinations is still missing. This is, at least in part, due to major problems to express and purify recombinant IL-35^13,15^ and general lack of recombinant IL-39.

Therefore, we decided to analyze signal transduction of the described IL-35 and potential IL-39 receptor complexes using synthetic cytokine receptors. Moreover, the synthetic cytokine receptors were used to generate additional thus far not described receptor complexes within the IL-12-type cytokine family. The synthetic cytokine receptors had a modular composition with the extracellular/membrane region (EXR) of one receptor fused to the intracellular region (IR) of another receptor, thereby enabling extracellular receptor activation by IL-12 or IL-23. In total we generated six synthetic receptor chimeras: IL-12Rβ2_EXR_-gp130_IR_, IL-12Rβ1_EXR_-gp130_IR_, IL-23R_EXR_-gp130_IR_, IL-12Rβ1_EXR_-IL-12Rβ2_IR_, IL-12Rβ1_EXR_-IL-23R_IR_, and IL-12Rβ1_EXR_-WSX-1_IR_ (Fig. 1B). We verified the cell surface localization and expression of all synthetic receptor chimeras in Ba/F3 cells by flow cytometry and Western blotting (Fig. S2A,B). All receptor chimeras with the extracellular domains of IL-12Rβ1 and IL-12Rβ2 were as well expressed on the cell surface as their wild-type IL-12Rβ1 and IL-12Rβ2 receptors. The IL-23R_EXR_-gp130_IR_ variant was, however, much lower expressed on the cell surface as IL-23R. As cellular model system we used Ba/F3 cells to analyze signal transduction of synthetic IL-6/IL-12-type cytokine receptors. As described previously, Ba/F3 cells, stably expressing IL-12Rβ1:IL-12Rβ2 or IL-12Rβ1:IL-23R^18,19^, stimulated with IL-12 and IL-23 induced STAT1, STAT3 and Erk1/2 phosphorylation and cytokine-dependent cellular proliferation after stimulation with IL-12 and IL-23, respectively (Fig. S3A-D). The transcription of the gene Pim-1 has been shown to be dependent on STAT3-activation^20^. Consequently, activation of IL-12 signal transduction resulted in the transcription of the STAT3 target gene Pim-1 (Fig. S2E). STAT4 phosphorylation was not analyzed, because STAT4 is not expressed in Ba/F3 cells^21^. Ba/F3 cells were stably transduced with a cDNA coding for human gp130 and responsive to Hyper-IL-6. Hyper-IL-6 (HIL-6) is a fusion protein of IL-6 and soluble IL-6R connected via a flexible peptide linker specifically inducing IL-6-trans-signaling via gp130^17^. Using these cells, Hyper-IL-6-induced proliferation and Pim-1 mRNA expression was observed for all cell lines and served as internal control (Fig. S3A,B,D,E). For induction of synthetic signal transduction the appropriate Hyper-cytokines for IL-12 and IL-23, HIL-12 and HIL-23, have been generated and expressed^18,19^ (Fig. S3F).

Since the expression of IL-23R_EXR_-gp130_IR_ was reduced as compared to IL-23R, we initially analyzed if this receptor chimera was able to induce sustained synthetic signal transduction. As a model system, we tested the functional assembly of intracellular regions of IL-27-signaling by IL-23 stimulation of IL-12Rβ1_EXR_-WSX-1_IR_ and IL-23R_EXR_-gp130_IR_ in stably transduced Ba/F3 cells (Fig. 1C,D). We compared the dose-response proliferation curves of these Ba/F3 cells after IL-23 stimulation with Ba/F3 cells expressing wild-type IL-23R and IL-12Rβ1. Our results showed that the synthetic WSX-1/gp130-heterodimer was about 10fold less sensitive to IL-23 stimulation as compared to canonical IL-12Rβ1/IL-23R activation (Fig. 1E). Moreover, stimulation with higher amounts of IL-23 (125 and 250 ng/ml) induced only minimal STAT1/3 phosphorylation, whereas Erk1/2 phosphorylation was not detectable (Fig. 1F). Our results revealed that this synthetic receptor combination conferred only reduced biological activity. Therefore we decided to use IL-12-inducible chimeric receptors to analyze IL-35 signaling.

### The synthetic IL-35 receptor complexes consisting of IL-12Rβ2 and gp130 are biologically active

Next, we used our synthetic receptor chimeras to generate receptor combinations, which will be activated by IL-12 to induce IL-35 signal transduction.

Three signal transducing complexes for IL-35 have been described by Collison *et al*., a hetero-dimeric complex consisting of IL-12Rβ2/gp130 and two homo-dimeric receptor complexes consisting of IL-12Rβ2/IL-12Rβ2 and gp130/gp130^11^. IL-12Rβ2 is involved in IL-12-induced and gp130 is involved in IL-6-induced signal transduction and result in phosphorylation of STAT1, 3, 4 and STAT1, 3, 5, respectively. Interestingly, the signal transducing complexes for IL-35 were described to specifically induce STAT1- and STAT4-phosphorylation for IL-12Rβ2/gp130, only STAT4-phosphorylation for IL-12Rβ2/IL-12Rβ2 and only STAT1-phosphorylation for gp130/gp130. To date, Erk1/2 phosphorylation was not analyzed for IL-35^11^.

**Figure 2:**
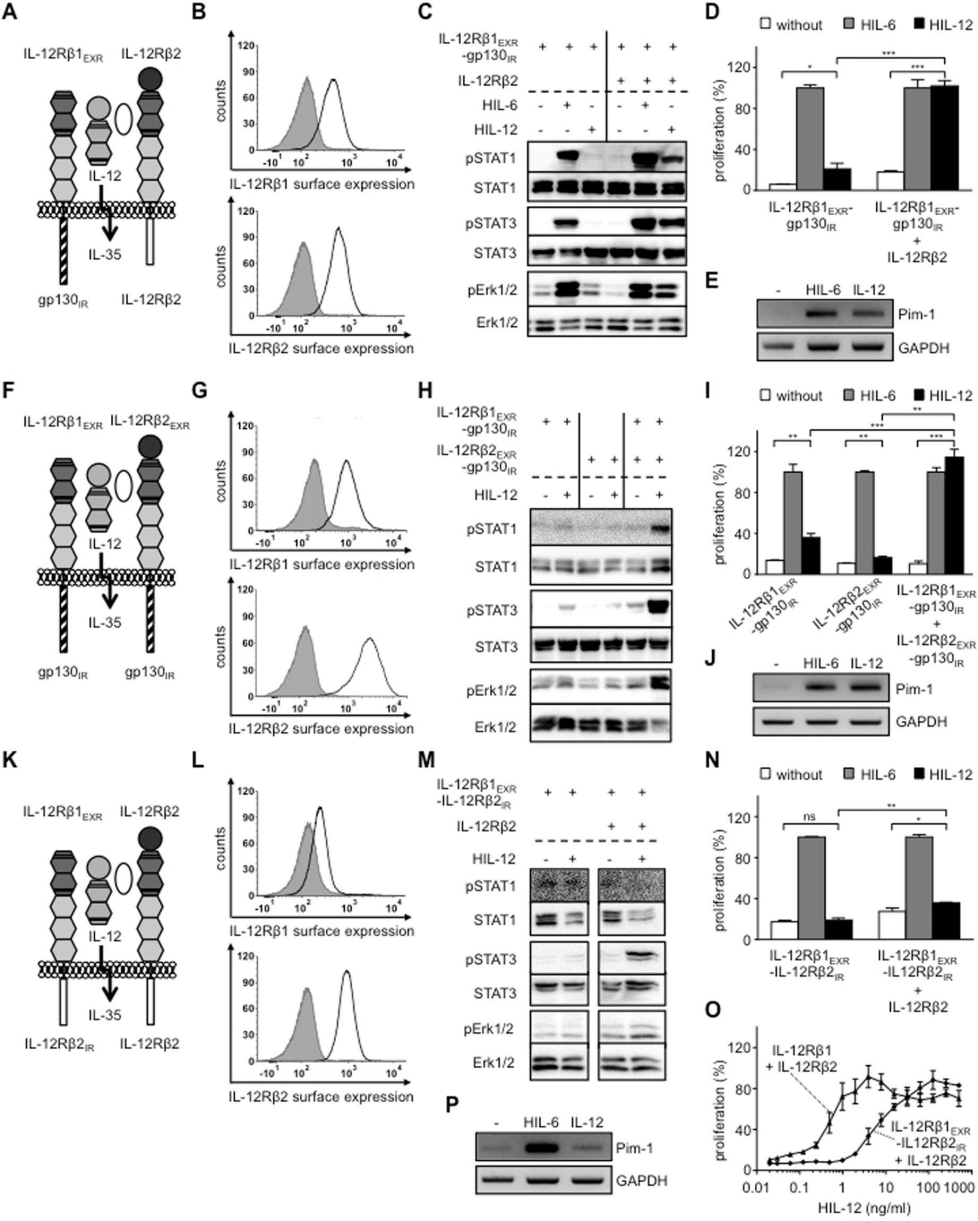
Synthetic IL-35 receptor complexes consisting of IL-12Rβ2 and gp130 are biologically active. (**A**) Schematic overview of IL-35-type signaling by IL-12-induced receptor activation of IL-12Rβ1_EXR_-gp130_IR_ and IL-12Rβ2. (**B**) Representative histograms of IL-12Rβ1_EXR_-gp130_IR_ (upper panel) and IL-12Rβ2 (lower panel) surface expression of Ba/F3-gp130/IL-12Rβ1_EXR_-gp130_IR_/IL-12Rβ2 cells (light solid lines). Gray-shaded areas indicate Ba/F3-gp130 cells (negative control). (**C**) Analysis of STAT1/3 and Erk1/2 activation. Ba/F3-gp130/IL-12Rβ1_EXR_-gp130_IR_ and Ba/F3-gp130/IL-12Rβ1_EXR_-gp130_IR_/IL-12Rβ2 cells were washed three times, starved, and stimulated with 4 ng/ml HIL-12 for 30 min or 10 ng/ml HIL-6. Cellular lysates were prepared, and equal amounts of total protein (50 μg/lane) were loaded on SDS gels, followed by immunoblotting using specific antibodies for phospho-STAT1/3/Erk1/2 and STAT1/3/Erk1/2. Western blot data show one representative experiment out of three. (**D**) Cellular proliferation of Ba/F3-gp130/IL-12Rβ1_EXR_-gp130_IR_ and Ba/F3-gp130/IL-12Rβ1_EXR_-gp130_IR_/IL-12Rβ2 cells. Equal numbers of cells were cultured for 3 days in the presence of 4 ng/ml HIL-12. Proliferation was measured using the colorimetric CellTiter-Blue Cell Viability Assay. HIL-6–induced proliferation (10 ng/ml) was set to 100%. One representative experiment out of three is shown. Error bars represent SD. Statistical analysis used a Welch t test (n = 3; *p ≤ 0.05; ***p ≤ 0.001). **(E)** Analysis of STAT3 target gene expression of Pim-1 in Ba/F3-gp130/IL-12Rβ1_EXR_-gp130_IR_/IL-12Rβ2 cells stimulated with 4 ng/ml IL-12 for 2 h. One representative experiment out of two is shown. (**F**) Schematic overview of IL-35-type signaling by IL-12-induced receptor activation of IL-12Rβ1_EXR_-gp130_IR_ and IL-12Rβ2_EXR_-gp130_IR_. (**G**) Representative histograms of IL-12Rβ1_EXR_-gp130_IR_ (upper panel) and IL-12Rβ2_EXR_-gp130_IR_ (lower panel) surface expression of Ba/F3-gp130/IL-12Rβ1_EXR_-gp130_IR_/IL-12Rβ2_EXR_-gp130_IR_ cells (light solid lines). Gray-shaded areas indicate Ba/F3-gp130 cells (negative control). (**H**) Analysis of STAT1/3 and Erk1/2 activation of Ba/F3-gp130/IL-12Rβ1_EXR_-gp130_IR_, Ba/F3-gp130/IL-12Rβ2_EXR_-gp130_IR_ and Ba/F3-gp130/IL-12Rβ1_EXR_-gp130_IR_/IL-12Rβ2_EXR_-gp130_IR_ cells as described in (**C**). Western blot data show one representative experiment out of three. (**I**) Cellular proliferation of Ba/F3-gp130/IL-12Rβ1_EXR_-gp130_IR_, Ba/F3-gp130/IL-12Rβ2_EXR_-gp130_IR_ and Ba/F3-gp130/IL-12Rβ1_EXR_-gp130_IR_/IL-12Rβ2_EXR_-gp130_IR_ cells as described in (**D**). One representative experiment out of three is shown. Error bars represent SD. Statistical analysis used a Welch t test (n = 3; **p ≤ 0.01; ***p ≤ 0.001). **(J)** Analysis of STAT3 target gene expression of Pim-1 in Ba/F3-gp130/IL-12Rβ1_EXR_-gp130_IR_/IL-12Rβ2_EXR_-gp130_IR_ cells stimulated with 4 ng/ml IL-12 for 2 h. One representative experiment out of two is shown. (**K**) Schematic overview of IL-35-type signaling by IL-12-induced receptor activation of IL-12Rβ1_EXR_-IL-12Rβ2_IR_ and IL-12Rβ2. (**L**) Representative histograms of IL-12Rβ1_EXR_-IL-12Rβ2_IR_ (upper panel) and IL-12Rβ2 (lower panel) surface expression of Ba/F3-gp130/IL-12Rβ1_EXR_-IL-12Rβ2_IR_/IL-12Rβ2 cells (light solid lines). Gray-shaded areas indicate Ba/F3-gp130 cells (negative control). (**M**) Analysis of STAT1/3 and Erk1/2 activation of Ba/F3-gp130/IL-12Rβ1_EXR_-IL-12Rβ2_IR_ and Ba/F3-gp130/IL-12Rβ1_EXR_-IL-12Rβ2_IR_/IL-12Rβ2 cells as described in (**C**). Western blot data show one representative experiment out of three. (**N**) Cellular proliferation of Ba/F3-gp130/IL-12Rβ1_EXR_-IL-12Rβ2_IR_ and Ba/F3-gp130/IL-12Rβ1_EXR_-IL-12Rβ2_IR_/IL-12Rβ2 cells as described in (**D**). One representative experiment out of three is shown. Error bars represent SD. Statistical analysis used a Welch t test (n = 3; ns = not significant; *p ≤ 0.05; **p ≤ 0.01). (**O**) Cellular proliferation of Ba/F3-gp130/IL-12Rβ1_EXR_-IL-12Rβ2_IR_/IL-12Rβ2 and Ba/F3-gp130/IL-12Rβ1/IL-12Rβ2 cells. Equal numbers of cells were cultured for 3 days in the presence of HIL-12 (0.01 to 500 ng/ml). Proliferation was measured using the colorimetric CellTiter-Blue Cell Viability Assay. HIL-6–induced proliferation (10 ng/ml) was set to 100%. One representative experiment out of two is shown. Error bars represent SD. **(P)** Analysis of STAT3 target gene expression of Pim-1 in Ba/F3-gp130/IL-12Rβ1_EXR_-IL-12Rβ2_IR_/IL-12Rβ2 cells stimulated with 4 ng/ml IL-12 for 2 h. One representative experiment out of two is shown.

First of all, we generated Ba/F3 cells, stably expressing the synthetic receptor chimeras IL-12Rβ1_EXR_-gp130_IR_ with IL-12Rβ2 for analysis of the IL-35 receptor complex gp130/IL-12Rβ2 (Fig. 2A,B). Stimulation of Ba/F3-IL-12Rβ1_EXR_-gp130_IR_/IL-12Rβ2 cells with IL-12 resulted in STAT1, 3 and Erk1/2 phosphorylation and cytokine-dependent proliferation, whereas Ba/F3 cells expressing only one receptor chain were not activated (Fig. 2C,D). IL-12-induced IL-12Rβ1_EXR_-gp130_IR_/IL-12Rβ2 signal transduction resulted in the transcription of the STAT3 target gene Pim-1 (Fig. 2E). Next, we generated Ba/F3 cells, stably expressing the synthetic receptor chimeras IL-12Rβ1_EXR_-gp130_IR_ and IL-12Rβ2_EXR_-gp130_IR_ representing the IL-35 receptor complex gp130/gp130 (Fig. 2F,G). Stimulation of Ba/F3-IL-12Rβ1_EXR_-gp130_IR_/IL-12Rβ2_EXR_-gp130_IR_ cells with IL-12 induced STAT1, 3 and Erk1/2 phosphorylation and cytokine-dependent proliferation (Fig. 2H,I). Again, Ba/F3 cells expressing only one receptor chain were not activated (Fig. 2H,I). Also IL-12-induced IL-12Rβ1_EXR_-gp130_IR_/IL-12Rβ2_EXR_-gp130_IR_ signal transduction resulted in the transcription of the STAT3 target gene Pim-1 (Fig. 2J). Finally, Ba/F3 cells, stably expressing IL-12Rβ1_EXR_-IL-12Rβ2_IR_ and IL-12Rβ2, representative for the IL-35 receptor complex IL-12Rβ2/IL-12Rβ2 (Fig. 2K,L) were analyzed for signal transduction and proliferation after IL-12 stimulation. IL-12 stimulation induced STAT3 and weak Erk1/2 phosphorylation, whereas STAT1 phosphorylation was not detectable (Fig. 2M). Since standard concentrations of 4 ng/ml IL-12 did not induce sustained cellular proliferation of the respective Ba/F3 cells (Fig. 2N), we compared dose-response proliferation curves of Ba/F3-IL-12Rβ1_EXR_-IL-12Rβ2_IR_/IL-12Rβ2 and Ba/F3-IL-12Rβ1/IL-12Rβ2 cells after IL-12 stimulation. Our results showed that the synthetic IL-12Rβ2-homodimer was about 10fold less sensitive to IL-12 stimulation as compared to canonical IL-12Rβ1/IL-12Rβ2 activation (Fig. 2O), suggesting that either our synthetic fusion protein conferred reduced biological activity or homo-dimers of IL-12Rβ2 were per se less effective as compared to canonical IL-12 signaling. This was reflected for Pim-1, which was only slightly increased above back-ground level after IL-12-induced IL-12Rβ1_EXR_-IL-12Rβ2_IR_ and IL-12Rβ2 signal transduction (Fig. 2P).

Taken together, our combinatorial approach demonstrated that hetero-dimerization of the intracellular regions of IL-12Rβ2/gp130, gp130/gp130 and IL-12Rβ2/IL-12Rβ2 which represent three out of four described IL-35 cytokine receptor combinations resulted in receptor activation. At least in Ba/F3 cells, signal transduction of these receptor combinations was more canonical and not restricted to STAT1 and STAT4.

### IL-12-stimulation induced intracellular signaling of synthetic receptors mimicking the IL-35 receptor complex IL-12Rβ2:WSX-1

**Figure 3:**
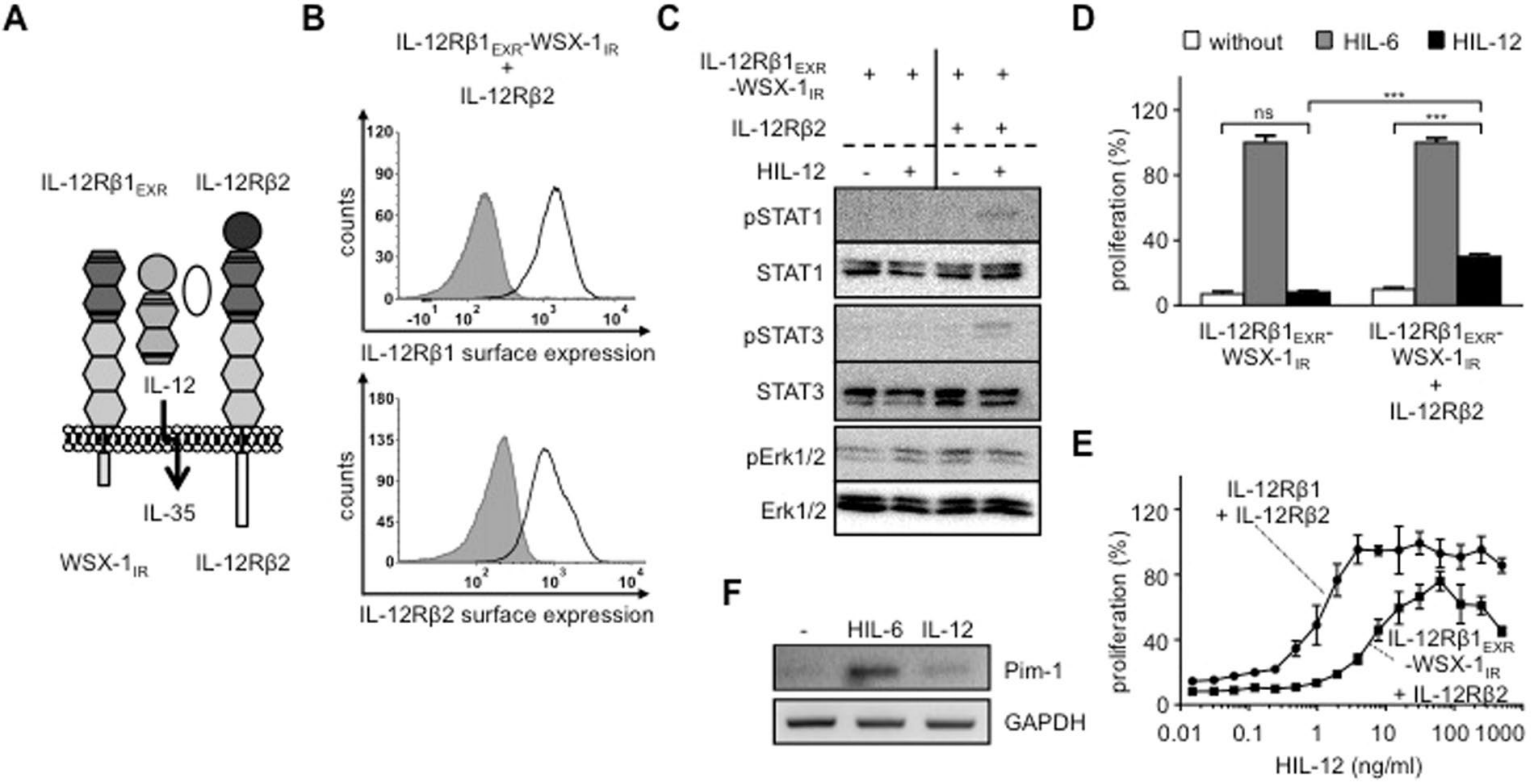
IL-12-stimulation induced intracellular signaling of synthetic receptors mimicking the IL-35 receptor complex IL-12Rβ2:WSX-1. (**A**) Schematic overview of IL-35-type signaling by IL-12-induced receptor activation of IL-12Rβ1_EXR_-WSX-1_IR_ and IL-12Rβ2. (**B**) Representative histograms of IL-12Rβ1_EXR_-WSX-1_IR_ (upper panel) and IL-12Rβ2 (lower panel) surface expression of Ba/F3-gp130/IL-12Rβ1_EXR_-WSX-1_IR_/IL-12Rβ2 cells (light solid lines). Gray-shaded areas indicate Ba/F3-gp130 cells (negative control). (**C**) Analysis of STAT1/3 and Erk1/2 activation. Ba/F3-gp130/IL-12Rβ1_EXR_-WSX-1_IR_ and Ba/F3-gp130/IL-12Rβ1_EXR_-WSX-1_IR_/IL-12Rβ2 cells were washed three times, starved, and stimulated with 4 ng/ml HIL-12 for 30 min. Cellular lysates were prepared, and equal amounts of total protein (50 μg/lane) were loaded on SDS gels, followed by immunoblotting using specific antibodies for phospho-STAT1/3/Erk1/2 and STAT1/3/Erk1/2. Western blot data show one representative experiment out of three. (**D**) Cellular proliferation of Ba/F3-gp130/IL-12Rβ1_EXR_-WSX-1_IR_ and Ba/F3-gp130/IL-12Rβ1_EXR_-WSX-1_IR_/IL-12Rβ2 cells. Equal numbers of cells were cultured for 3 days in the presence of 4 ng/ml HIL-12. Proliferation was measured using the colorimetric CellTiter-Blue Cell Viability Assay. HIL-6–induced proliferation (10 ng/ml) was set to 100%. One representative experiment out of three is shown. Error bars represent SD. Statistical analysis used a Welch t test (n = 3; ns = not significant; ***p ≤ 0.001). (**E**) Cellular proliferation of Ba/F3-gp130/IL-12Rβ1_EXR_-WSX-1_IR_/IL-12Rβ2 and Ba/F3-gp130/IL-12Rβ1/IL-12Rβ2 cells. Equal numbers of cells were cultured for 3 days in the presence of HIL-12 (0.01 to 500 ng/ml). Proliferation was measured using the colorimetric CellTiter-Blue Cell Viability Assay. HIL-6–induced proliferation (10 ng/ml) was set to 100%. One representative experiment out of four is shown. Error bars represent SD. **(F)** Analysis of STAT3 target gene expression of Pim-1 in Ba/F3-gp130/IL-12Rβ1_EXR_-WSX-1_IR_/IL-12Rβ2 cells stimulated with 4 ng/ml IL-12 for 2 h. One representative experiment out of two is shown.

Wang *et al*. described a fourth receptor complex for IL-35 in regulatory B cells, promoting the differentiation of B cells into a B_reg_ subset that produces IL-10 and IL-35. IL-35 signaling in B cells was mediated by IL-12Rβ2 and WSX-1 and resulted in STAT1, 3 and 5 phosphorylation. IL-35 also activates T cells where it induced STAT1, 3 and 4 phosphorylation. STAT5 and STAT6 phosphorylation was not detected^13^. To engineer this receptor complex, we generated Ba/F3 cells, stably expressing IL-12Rβ2 and IL-12Rβ1_EXR_-WSX-1_IR_ (IL-35 for IL-12Rβ2/WSX-1) (Fig. 3A,B). Stimulation of Ba/F3-IL-12Rβ1_EXR_-WSX-1_IR_/IL-12Rβ2 cells with IL-12 resulted in STAT1 and 3 phosphorylation. Erk1/2 phosphorylation was, however, not detected (Fig. 3C). Again, standard concentrations of 4 ng/ml IL-12 induced only minimal cellular proliferation of Ba/F3-IL-12Rβ1_EXR_-WSX-1_IR_/IL-12Rβ2 cells (Fig. 3D). Therefore, we compared the dose-response proliferation curves of Ba/F3-IL-12Rβ1_EXR_-WSX-1_IR_/IL-12Rβ2 and Ba/F3-IL-12Rβ1/IL-12Rβ2 cells after IL-12 stimulation. Our results showed that the synthetic IL-12Rβ2/WSX-1-heterodimer was about 10fold less sensitive to IL-12 stimulation as compared to canonical IL-12Rβ1/IL-12Rβ2 activation (Fig. 3E), suggesting that also these synthetic fusion proteins conferred reduced biological activity or hetero-dimers of IL-12Rβ2/WSX-1 were per se less effective as compared to canonical IL-12 signaling. This was also reflected for Pim-1, which was only slightly increased above back-ground level after IL-12-induced IL-12Rβ1_EXR_-WSX-1_IR_/IL-12Rβ2 signal transduction (Fig. 2F).

Our results showed, that also hetero-dimerization of the intracellular regions of IL-12Rβ2 and WSX-1 which represents the fourth described IL-35 cytokine receptor complex resulted in signal transduction. As described previously, signal transduction of this receptor combination was canonical.

### Analysis of IL-12- and IL-23-stimulation of synthetic receptor complexes mimicking IL-39 and additional potential receptor complexes of the IL-12 family

Next, we used our synthetic cytokine receptor system to test the functional assembly of intracellular regions of IL-23R:gp130 for IL-39, IL-12Rβ1:gp130 for hypothetical IL-Y and IL-23R:IL-12Rβ2 for hypothetical IL-Z1 in Ba/F3 cells.

**Figure 4:**
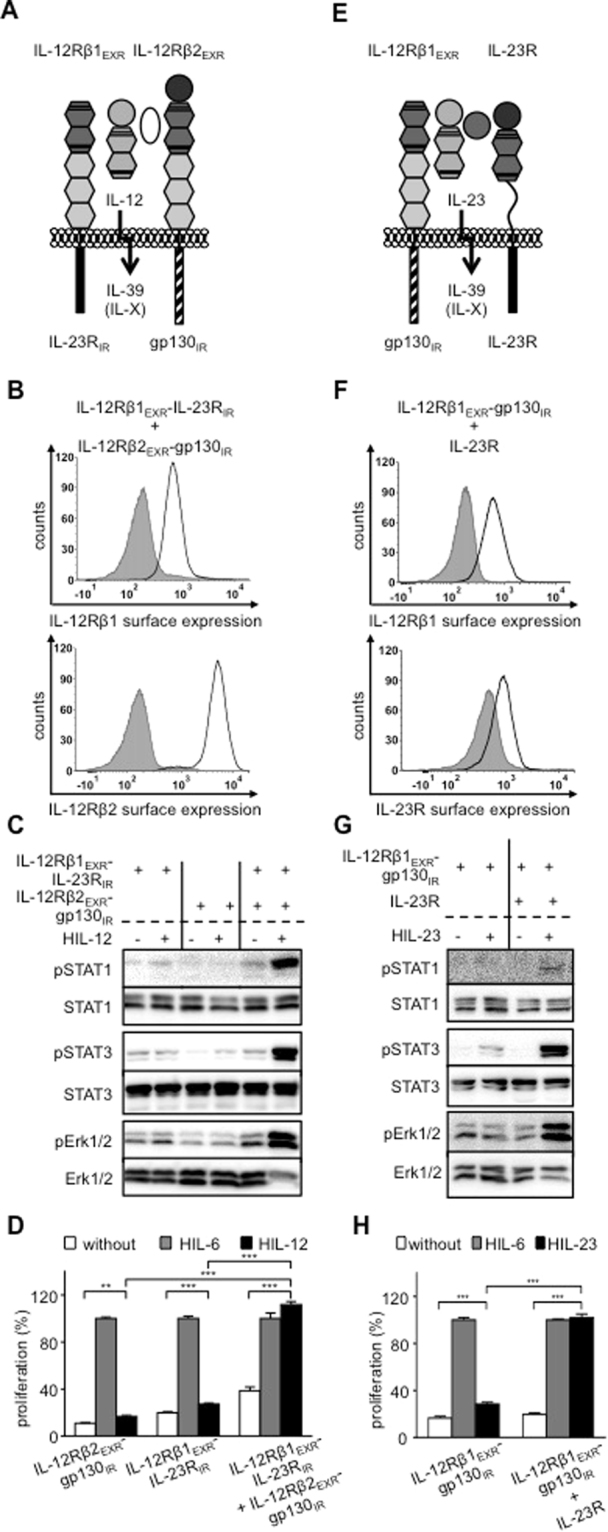
Analysis of IL-12- and IL-23-stimulation of synthetic receptor complexes mimicking IL-23R:gp130 (IL-39). (**A**) Schematic overview of IL-39-type signaling by IL-12-induced receptor activation of IL-12Rβ1_EXR_-IL-23R_IR_ and IL-12Rβ2_EXR_-gp130_IR_. (**B**) Representative histograms of IL-12Rβ1_EXR_-IL-23R_IR_ (upper panel) and IL-12Rβ2_EXR_-gp130_IR_ (lower panel) surface expression of Ba/F3-gp130/IL-12Rβ1_EXR_-IL-23R_IR_/IL-12Rβ2_EXR_-gp130_IR_ cells (light solid lines). Gray-shaded areas indicate Ba/F3-gp130 cells (negative control). (**C**) Analysis of STAT1/3 and Erk1/2 activation. Ba/F3-gp130/IL-12Rβ1_EXR_-IL-23R_IR_, Ba/F3-gp130/IL-12Rβ2_EXR_-gp130_IR_ and Ba/F3-gp130/IL-12Rβ1_EXR_-IL-23R_IR_/IL-12Rβ2_EXR_-gp130_IR_ cells were washed three times, starved, and stimulated with 4 ng/ml HIL-12 for 30 min. Cellular lysates were prepared, and equal amounts of total protein (50 μg/lane) were loaded on SDS gels, followed by immunoblotting using specific antibodies for phospho-STAT1/3/Erk1/2 and STAT1/3/Erk1/2. Western blot data show one representative experiment out of three. (**D**) Cellular proliferation of Ba/F3-gp130/IL-12Rβ1_EXR_-IL-23R_IR_, Ba/F3-gp130/IL-12Rβ2_EXR_-gp130_IR_ and Ba/F3-gp130/IL-12Rβ1_EXR_-IL-23R_IR_/IL-12Rβ2_EXR_-gp130_IR_ cells. Equal numbers of cells were cultured for 3 days in the presence of 4 ng/ml HIL-12. Proliferation was measured using the colorimetric CellTiter-Blue Cell Viability Assay. HIL-6–induced proliferation (10 ng/ml) was set to 100%. One representative experiment out of three is shown. Error bars represent SD. Statistical analysis used a Welch t test (n = 3; **p ≤ 0.01; ***p ≤ 0.001). (**E**) Schematic overview of IL-39-type signaling by IL-23-induced receptor activation of IL-12Rβ1_EXR_-gp130_IR_ and IL-23R. (**F**) Representative histograms of IL-12Rβ1_EXR_-gp130_IR_ (upper panel) and IL-23R (lower panel) surface expression of Ba/F3-gp130/IL-12Rβ1_EXR_-gp130_IR_/IL-23R cells (light solid lines). Gray-shaded areas indicate Ba/F3-gp130 cells (negative control). (**G**) Analysis of STAT1/3 and Erk1/2 activation of Ba/F3-gp130/IL-12Rβ1_EXR_-gp130_IR_ and Ba/F3-gp130/IL-12Rβ1_EXR_-gp130_IR_/IL-23R cells as described in (**C**). Western blot data show one representative experiment out of three. (**H**) Cellular proliferation of Ba/F3-gp130/IL-12Rβ1_EXR_-gp130_IR_ and Ba/F3-gp130/IL-12Rβ1_EXR_-gp130_IR_/IL-23R cells as described in (**D**). One representative experiment out of three is shown. Error bars represent SD. Statistical analysis used a Welch t test (n = 3; ***p ≤ 0.001).

IL-39 induced STAT1 and STAT3 phosphorylation but not STAT4 and STAT5 phosphorylation in primary murine B cells. However, Erk1/2 phosphorylation was not analyzed^6,22^. IL-39 was recently suggested to signal via a heterodimer of IL-23R:gp130^9^. Ba/F3 cells stably expressing IL-12Rβ1_EXR_-IL-23R_IR_ and IL-12Rβ2_EXR_-gp130_IR_, representative for the potential IL-39 receptor complex IL-23R:gp130 for IL-39 (Fig. 4A,B) were stimulated with IL-12. We detected sustained STAT1, 3 and Erk1/2-phosphorylation and cytokine-dependent proliferation (Fig. 4C,D). Albeit, IL-23-induced signaling appeared to be less suitable (compare Fig. 1B-E), we verified our results using IL-23-dependent synthetic cytokine receptors. Ba/F3 cells stably expressing IL-12Rβ1 and IL-23R_EXR_-gp130_IR_ (Fig. 4E,F) stimulated with IL-23 also showed sustained STAT3 and Erk1/2-phosphorylation and cytokine-dependent proliferation, whereas STAT1 phosphorylation was much lower as compared to the respective IL-12-inducible receptor combination (Fig. 4G-H). The data presented here, revealed that IL-23R and gp130 which is the proposed IL-39 receptor complex can execute signal transduction.

**Figure 5:**
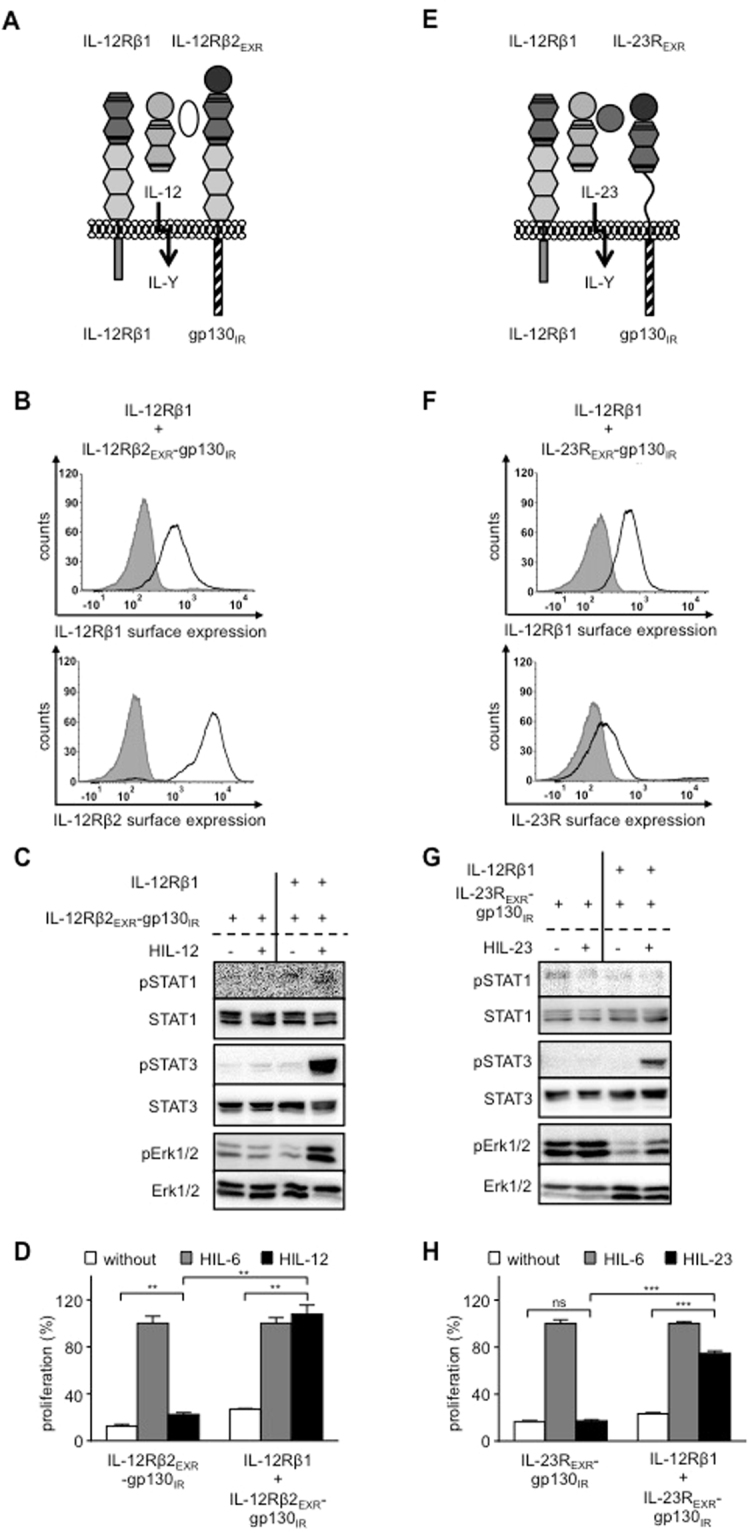
Analysis of IL-12- and IL-23-stimulation of synthetic receptor complexes mimicking IL-12Rβ1:gp130. (**A**) Schematic overview of IL-Y-type signaling by IL-12-induced receptor activation of IL-12Rβ1 and IL-12Rβ2_EXR_-gp130_IR_. (**B**) Representative histograms of IL-12Rβ1 (upper panel) and IL-12Rβ2_EXR_-gp130_IR_ (lower panel) surface expression of Ba/F3-gp130/IL-12Rβ1/IL-12Rβ2_EXR_-gp130_IR_ cells (light solid lines). Gray-shaded areas indicate Ba/F3-gp130 cells (negative control). (**C**) Analysis of STAT1/3 and Erk1/2 activation. Ba/F3-gp130/IL-12Rβ2_EXR_-gp130_IR_ and Ba/F3-gp130/IL-12Rβ1/IL-12Rβ2_EXR_-gp130_IR_ cells were washed three times, starved, and stimulated with 4 ng/ml HIL-12 for 30 min. Cellular lysates were prepared, and equal amounts of total protein (50 μg/lane) were loaded on SDS gels, followed by immunoblotting using specific antibodies for phospho-STAT1/3/Erk1/2 and STAT1/3/Erk1/2. Western blot data show one representative experiment out of three. (**D**) Cellular proliferation of Ba/F3-gp130/IL-12Rβ2_EXR_-gp130_IR_ and Ba/F3-gp130/IL-12Rβ1/IL-12Rβ2_EXR_-gp130_IR_ cells. Equal numbers of cells were cultured for 3 days in the presence of 4 ng/ml HIL-12. Proliferation was measured using the colorimetric CellTiter-Blue Cell Viability Assay. HIL-6–induced proliferation (10 ng/ml) was set to 100%. One representative experiment out of three is shown. Error bars represent SD. Statistical analysis used a Welch t test (n = 3; **p ≤ 0.01). (**E**) Schematic overview of IL-Y-type signaling by IL-12-induced receptor activation of IL-12Rβ1 and IL-23R_EXR_-gp130_IR_. (**F**) Representative histograms of IL-12Rβ1 (upper panel) and IL-23R_EXR_-gp130_IR_ (lower panel) surface expression of Ba/F3-gp130/IL-12Rβ1/IL-23R_EXR_-gp130_IR_ cells (light solid lines). Gray-shaded areas indicate Ba/F3-gp130 cells (negative control). (**G**) Analysis of STAT1/3 and Erk1/2 activation of Ba/F3-gp130/IL-23_EXR_-gp130_IR_ and Ba/F3-gp130/IL-12Rβ1/IL-23_EXR_-gp130_IR_ cells as described in (**C**). Western blot data show one representative experiment out of three. (**H**) Cellular proliferation of Ba/F3-gp130/IL-23_EXR_-gp130_IR_ and Ba/F3-gp130/IL-12Rβ1/IL-23_EXR_-gp130_IR_ cells as described in (**D**). One representative experiment out of two is shown. Error bars represent SD. Statistical analysis used a Welch t test (n = 3; ns = not significant; ***p ≤ 0.001).

Ba/F3 cells stably expressing IL-12Rβ1 and IL-12Rβ2_EXR_-gp130_IR,_ representative for the potential IL-Y receptor complex IL-12Rβ1:gp130, (Fig. 5A,B) were stimulated with IL-12 and showed STAT3 and Erk1/2 phosphorylation and cytokine-dependent proliferation (Fig. 5C,D), demonstrating that IL-12Rβ1 and gp130 can form a biological active heterodimer. STAT1 phosphorylation was, however, only hardly detectable (lower band in Fig. 5C). Again, we verified our results using IL-23-dependent synthetic cytokine receptors. Ba/F3 cells stably expressing IL-12Rβ1 and IL-23R_EXR_-gp130_IR_ (Fig. 5E,F) were stimulated with IL-23 also showed sustained STAT3 and Erk1/2-phosphorylation and cytokine-dependent proliferation (Fig. 5G,H). Here, STAT1 phosphorylation was not detectable (Fig. 5G). Our results demonstrate that the combination of the intracellular domains of IL-12Rβ1 and gp130 can induce cellular signal transduction.

**Figure 6:**
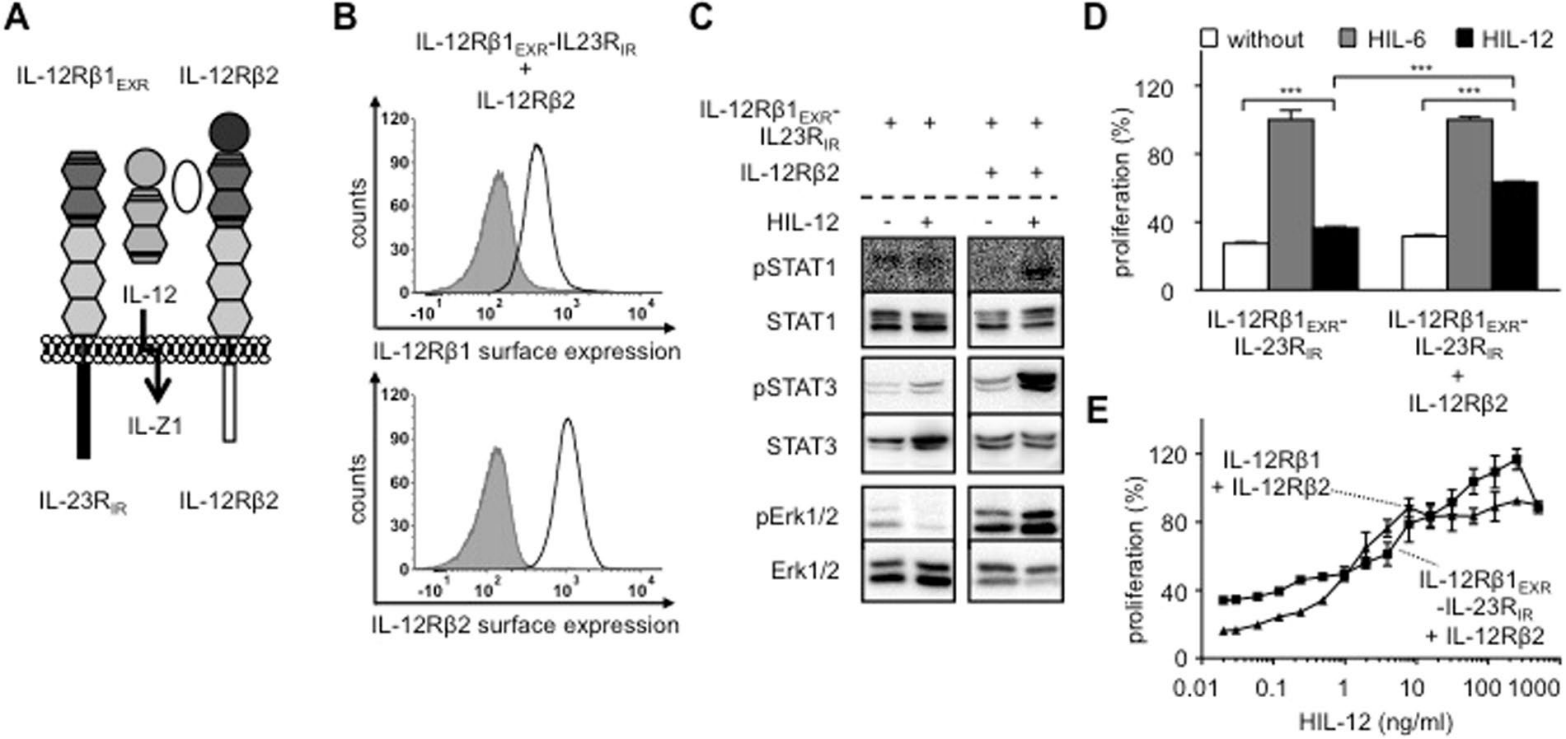
Analysis of IL-12-stimulation of synthetic receptor complexes mimicking IL-23R:IL-12Rβ2. (**A**) Schematic overview of IL-Z-type signaling by IL-12-induced receptor activation of IL-12Rβ1_EXR_-IL-23R_IR_ and IL-12Rβ2. (**B**) Representative histograms of IL-12Rβ1_EXR_-IL-23R_IR_ (upper panel) and IL-12Rβ2 (lower panel) surface expression of Ba/F3-gp130/IL-12Rβ1_EXR_-IL-23R_IR_/IL-12Rβ2 cells (light solid lines). Gray-shaded areas indicate Ba/F3-gp130 cells (negative control). (**C**) Analysis of STAT1/3 and Erk1/2 activation. Ba/F3-gp130/IL-12Rβ1_EXR_-IL-23R_IR_ and Ba/F3-gp130/IL-12Rβ1_EXR_-IL-23R_IR_/IL-12Rβ2 cells were washed three times, starved, and stimulated with 4 ng/ml HIL-12 for 30 min. Cellular lysates were prepared, and equal amounts of total protein (50 μg/lane) were loaded on SDS gels, followed by immunoblotting using specific antibodies for phospho-STAT1/3/Erk1/2 and STAT1/3/Erk1/2. Western blot data show one representative experiment out of three. (**D**) Cellular proliferation of Ba/F3-gp130/IL-12Rβ1_EXR_-IL-23R_IR_ and Ba/F3-gp130/IL-12Rβ1_EXR_-IL-23R_IR_/IL-12Rβ2 cells. Equal numbers of cells were cultured for 3 days in the presence of 4 ng/ml HIL-12. Proliferation was measured using the colorimetric CellTiter-Blue Cell Viability Assay. HIL-6–induced proliferation (10 ng/ml) was set to 100%. One representative experiment out of three is shown. Error bars represent SD. Statistical analysis used a Welch t test (n = 3; ***p ≤ 0.001). (**E**) Cellular proliferation of Ba/F3-gp130/IL-12Rβ1_EXR_-IL-23R_IR_/IL-12Rβ2 and Ba/F3-gp130/IL-12Rβ1/IL-12Rβ2 cells. Equal numbers of cells were cultured for 3 days in the presence of HIL-12 (0.01 to 500 ng/ml). Proliferation was measured using the colorimetric CellTiter-Blue Cell Viability Assay. HIL-6–induced proliferation (10 ng/ml) was set to 100%. One representative experiment out of two is shown. Error bars represent SD.

Finally, Ba/F3 cells stably expressing IL-12Rβ2 and IL-12Rβ1_EXR_-IL-23R_IR_, representative for the potential IL-Z1 receptor complex IL-12Rβ2:IL-23R (Fig. 6A,B) were stimulated with IL-12 and showed STAT1 (lower band), STAT3 and weak Erk1/2-phosphorylation and cytokine-dependent proliferation (Fig. 6C,D), which was also evident in comparison of dose-dependent IL-12-induced cellular proliferation (Fig. 6E), demonstrating that also IL-12Rβ2 and IL-23R form a biological active heterodimer.

In conclusion, our combinatorial approach demonstrated that the combination of the intracellular regions of all tested intracellular receptor regions within the IL-12 family resulted in signal transduction which represent known (IL-39) and potential cytokine receptor combinations which have not yet been described to be functionally addressed by a cytokine of the IL-6/IL-12 family.

## Discussion

In this study, we generated synthetic chimeric receptors of the IL-12/IL-6 type-cytokine family to mimic IL-35 and IL-39 signaling and to decipher additional potential receptor combinations within the IL-12 type cytokine family. Our receptor shuffling approach demonstrated that the IL-12 cytokine receptors are assembled as exchangeable, modular domain structures.

IL-35 is produced by forkhead box P3^+^ Treg cells and activated B cells and has important roles in preventing autoimmunity, maintaining self-tolerance, and suppressing antitumor immune responses^9^. Some difficulties with IL-35-induced signal transduction have emerged, since this cytokine cannot be produced in bacterial expression systems^15^ and only one group was able to produce very limited amounts in insect cells^13^. Secretion of IL-35 in eukaryotic cell culture supernatant was, however, not observed^15^. This was due to the retention of IL-35 and Hyper-IL-35 in the ER-golgi-system of producing cells. Other IL-6/IL-12-type Hyper-cytokines, such as Hyper-IL-6, Hyper-IL-12 and Hyper-IL-27 were secreted into cell culture supernatants and biologically active. Interestingly, even though *in vitro* reconstitution of biologically IL-12 was possible, using p40 from eukaryotic cells with p35 purified and refolded from *E*.*coli*, the same p35 did not form a biologically active complex with EBI3 obtained from eukaryotic cells^15^. This lack of recombinant, purified, biologically active cytokine has prevented the analysis of IL-35 signal transduction in typical cellular model systems, such as Ba/F3 cells^17,18^.

Here, the chimeric receptor strategy enabled the biologically active reconstitution of all described IL-35-induced receptor complexes, namely the two hetero-dimeric receptor complexes IL-12Rβ2/gp130 and IL-12Rβ2/WSX-1 and the two homo-dimeric receptor complexes of IL-12Rβ2/IL-12Rβ2 and gp130/gp130. Since the chimeric receptors contained the extracellular domains for IL-12Rβ1 and IL-12Rβ2, activation of IL-35 signaling was inducible by IL-12. The degree of STAT-activation between the individual cytokines seems to vary. Nevertheless, it has been clearly demonstrated that in principal all IL-6 and IL-12 family cytokines activate the same pattern of STAT proteins, mainly STAT1, STAT3, STAT4 and to a lesser extent also STAT5. The only known exception to date is IL-35. Previously, using primary T cells STAT1 and STAT4 phosphorylation was shown for IL-12Rβ2/gp130, only STAT4 phosphorylation for IL-12Rβ2/IL-12Rβ2 and only STAT1 phosphorylation for gp130/gp130, whereas Erk1/2 phosphorylation was not analyzed^11^. Homo-dimerization of gp130 without the need of a membrane-bound α-receptor such as IL-6R or IL-11R has besides IL-35 previously been observed for viral IL-6^23^ or IL-6 in complex with the soluble IL-6R^24^. However, in both cases phosphorylation of STAT1 and STAT3 was detected, whereas IL-35 solely activates STAT1^11^. Using Ba/F3 cells, STAT1, 3 and Erk1/2 phosphorylation was detected for gp130 containing receptor complexes IL-12Rβ2/gp130 and gp130/gp130. Interestingly, STAT3 phosphorylation, weak Erk1/2 phosphorylation and no STAT1 phosphorylation were detected for IL-12Rβ2 homo-dimers. IL-35 signaling in primary B cells was mediated by IL-12Rβ2 and WSX-1 and resulted in STAT1, 3 and 5 phosphorylation. IL-35 also activates primary T cells via IL-12Rβ2 and WSX-1 where it induced STAT1, 3 and 4 phosphorylation. STAT5 and STAT6 phosphorylation was not detected^13^. In Ba/F3 cells, however, the chimeric IL-35-mimicking IL-12Rβ2:WSX-1-receptor complex resulted in STAT1 and 3 phosphorylation but no Erk1/2-activation. Since Ba/F3 cells lack expression of STAT4, we cannot exclude that this might influence the signaling behavior of some chimeric receptors. Interestingly, STAT proteins appear to be at least to some extent interchangeable. The lack of STAT3 in murine embryonic fibroblasts shifted IL-6-signaling to an IFNγ-like response mediated by compensatory STAT1 activation^25^. The data presented here demonstrate that IL-35 signaling has the potential to induce almost canonical signaling as would be expected from a novel member of the IL-6/IL-12 type cytokine family. To this end, it is not clear why IL-35 induces different signaling patterns in the other studies^11,13^. One explanation might be that primary T and B cells have imprinted specialized signaling pathways. In a cellular model system, such as Ba/F3 cells, such imprinting is not present and the IL-35-mimicking receptors were able to execute more canonical signal transduction.

Recently, IL-39, which is composed of IL-23_p19 and EBI3, was supposed to induce signal transduction via IL-23R and gp130^9^. IL-39 is produced by activated B cells and induced differentiation and/or expansion of neutrophils, and contributes to lupus-like diseases in MRL/lpr mice^6,22^. Production of IL-39 by keratinocytes appeared to contribute to wound healing by dampening inflammatory responses^26^. The formation of IL-39 was, however, only demonstrated in culture supernatants by immune-precipitation^6^, and recombinant production and purification of biologically active IL-39 was not accomplished to date. Using chimeric receptors, the IL-23R:gp130 heterodimer was shown to induce STAT1 and STAT3 phosphorylation, which is on good agreement with the published STAT phosphorylation profiles of IL-39^6^. Interestingly, IL-12- and IL-23-induced IL-39 signaling resulted in comparable STAT3 but different STAT1 activation, which demonstrate that not only the intracellular domains determine the signaling strength but also the extracellular assembly. Recently a similar effect was described for the EPO-receptor. A point mutation in EPO was shown to reduce EPO receptor dimerization, which resulted in reduced STAT1 and STAT3 but not in reduced STAT5 activation^27^. In addition we also demonstrated Erk1/2 activation.

In a final step, two additional potential receptor combinations IL-12Rβ1:gp130 and IL-12Rβ2:IL-23R were shown to form biologically active receptor complexes, which might be activated by thus far unknown cytokines, IL-Y and IL-Z1, respectively. Albeit, we have not functionally tested the two remaining potential combinations of IL-6/IL-12 receptors, the WSX-1:IL-12Rβ1 alternative for IL-Y and WSX-1:IL-23R for IL-Z2, from our data we strongly assume that these combinations would also result in active receptor complexes.

In conclusion, all tested combinations of cytokine receptors of the IL-12 type cytokine family were biologically active, which illustrates the combinatory potential among this family. Interestingly, even though non-conventional STAT activation pattern were described for IL-35, we clearly demonstrate that these combinations induce more conventional STAT activation patterns.

## Methods

### Cells and reagents

COS-7 cells (ACC-60) were purchased from the Leibniz Institute DSMZ-German Collection of Microorganisms and Cell Cultures (Braunschweig, Germany). Murine Ba/F3-gp130 cells transduced with human gp130 were provided by Immunex (Seattle, WA, USA)^28^. The packaging cell line Phoenix-Eco was described previously^29^. Ba/F3-gp130 cell lines with murine IL-12Rβ1 and mIL-23R, as well as mIL-12Rβ1 and mIL-12Rβ2 (WT) were described previously^18,19^. All cell lines were grown in DMEM high glucose culture medium (GIBCO^®^, Thermo Fisher Scientific, Waltham, MA) supplemented with 10% fetal calf serum (GIBCO^®^, Thermo Fisher Scientific,), 60 mg/l penicillin and 100 mg/l streptomycin (Genaxxon bioscience GmbH, Ulm, Germany) at 37 °C with 5% CO_2_ in a water saturated atmosphere. Ba/F3-gp130 cells were maintained in the presence of Hyper-IL-6 (HIL-6), a fusion protein of IL-6 and the soluble IL-6R, which mimics IL-6 trans-signaling^17^. Either recombinant protein (10 ng/ml) or 0.2% of conditioned cell culture medium from a stable CHO-K1 clone secreting Hyper-IL-6 (final concentration 10 ng/ml as determined by ELISA) were used to supplement the growth medium. Ba/F3-gp130/IL-12Rβ1/IL-23R cells or variants thereof were stimulated with 0.2% of conditioned cell culture medium from a stable CHO-K1 clone secreting Hyper-IL-23 (HIL-23, a fusion of murine p40 and murine IL-23_p19) in a final concentration of 10 ng/ml, as determined by ELISA^18^. Ba/F3-gp130/IL-12Rβ1/IL-12Rβ2 cells or variants thereof were stimulated with 0.4% of conditioned cell culture medium from a stable CHO-K1 clone secreting Hyper-IL-12 (HIL-12, a fusion of murine p40 and murine IL-12_p35) in a final concentration of 4 ng/ml, as determined by ELISA^19^. Recombinant human IL-35-Fc (8608-IL) and murine IL-27 (2799-ML) were obtained from R&D Systems (Minneapolis, MN, USA). Phospho-STAT1 (Tyr701) (58D6), STAT1, phospho-STAT3 (Tyr705) (D3A7), STAT3 (124H6), phospho-p44/42 MAPK (Erk1/2) (Thr-202/Tyr-204) (D13.14.4E), p44/42 MAPK (Erk1/2) antibodies and myc-tag (71D10) rabbit mAb were obtained from Cell Signaling Technology (Frankfurt, Germany). Peroxidase-conjugated secondary mAbs were obtained from Pierce (Thermo Fisher Scientific, Waltham, MA). Phycoerythrin (PE) conjugated mIL-12Rβ1 and mIL-23R mAbs were from R&D Systems (Minneapolis, MN, USA). Purified hamster mIL-12Rβ2 and PE mouse Armenian and Syrian hamster IgG cocktail were purchased from BD Biosciences (Heidelberg, Germany). Alexa Fluor 647 conjugated Fab goat anti-rat IgG was obtained from Dianova (Hamburg, Germany).

### Cloning of murine IL-12 family receptors

To create chimeric receptors of the IL-6/IL-12 family, the intracellular parts of human gp130 (aa 642-918), murine IL-12Rβ2 (aa 659-874), murine IL-23R (aa 396-644) and murine WSX-1 (aa 532-623) were amplified by PCR from p409-myc-gp130^30^, pMOWS-puro-mIL-12Rβ2^19^, p409-mIL-23R^18^ or pMOWS-FUSIO^31^, and inserted into mIL-12Rβ1, mIL-12Rβ2 or mIL-23R p409 expression vectors^18,19^ where the respective coding sequences (mIL-12Rβ1: aa 592-738, mIL-12Rβ2: aa 659-874 or mIL-23R: aa 396-644) have been removed. A C-terminal c-myc tag was added. The cDNAs coding for the chimeric receptors were transferred into pMOWS-puro^29^ or pMOWS-hygro^31^ for retroviral transduction of Ba/F3-gp130 cells.

### Transfection, transduction and selection of cells

Ba/F3-gp130 cells were retrovirally transduced with the pMOWS expression plasmids coding for the various IL-23R, IL-12Rβ2 and IL-12Rβ1 variants as described^18^. Transduced cells were grown in standard DMEM medium as described above supplemented with 10 ng/ml HIL-6. Selection of transduced Ba/F3 cells was performed with puromycin (1.5 µg/ml) or hygromycin B (1 mg/ml) (Carl Roth GmbH, Karlsruhe, Germany) or both for at least two weeks. Afterwards, HIL-6 was washed away and the generated Ba/F3-gp130 cell lines were selected for HIL-23- and HIL-12-dependent growth.

### Cell viability assay

To remove the cytokines, Ba/F3-gp130 cell lines were washed 3 times with sterile PBS. 5 × 10^3^ cells were suspended in DMEM supplemented with 10% FCS, 60 mg/l penicillin and 100 mg/l streptomycin, and cultured for three days in a final volume of 100 µl with or without cytokines as indicated. The CellTiter-Blue^®^ Cell Viability Assay (Promega, Karlsruhe, Germany) was used to estimate the number of viable cells by recording the fluorescence (excitation 560 nm, emission 590 nm) using the Infinite M200 PRO plate reader (Tecan, Crailsheim, Germany) immediately after adding 20 µl of reagent per well (time point 0) and up to 2 h after incubation under standard cell culture conditions. The fluorescent signal from the CellTiter-Blue^®^ Reagent is proportional to the number of viable cells. All of the values were measured in triplicates per experiment. Fluorescence values were normalized by subtraction of time point 0 values. For direct comparison of the individual cell lines, proliferation in the presence of HIL-6 was defined as 100%. All experiments were performed at least two or three times, and one representative experiment was selected. Representative cell viability experiments were statistically analyzed using unpaired t test with Welch correction.

### Stimulation assays

For analysis of STAT1/3 and Erk1/2 activation in Ba/F3-gp130 cell lines, cells were starved for 4 h in serum-free medium. This was followed by stimulation with cytokines as indicated. Subsequently, cells were harvested and lysed in 50 mM Tris-HCl pH 7.5, 150 mM NaCl, 2 mM EDTA, 1 mM NaF, 1 mM Na_3_VO_4_, 1% Nonidet P-40 and 1% Triton X-100, supplemented with complete protease inhibitor cocktail tablets (Roche Diagnostics, Mannheim, Germany). Protein concentration of cell lysates was determined by BCA protein assay (Pierce, Thermo Scientific) according to the manufacturer’s instructions. Analysis of STAT1/3 and Erk1/2 activation was done by immunoblotting using 50 µg proteins from total cell lysates and detection with phospho-STAT1/3 or phospho-Erk1/2 mAbs and STAT1/3 or Erk1/2 mAbs. Total RNA was prepared and Pim-1 mRNA was analyzed by semi-quantitive RT-PCR with 25 cycles using the primers as described previously^20^.

### Western blotting

Defined amounts of proteins from cell lysates were loaded per lane, separated by SDS-PAGE under reducing conditions and transferred to PVDF membranes. The membranes were blocked in 5% fat-free dried skimmed milk in TBS-T (10 mM Tris-HCl pH 7.6, 150 mM NaCl, 1% Tween 20) and probed with the indicated primary antibodies in 5% fat-free dried skimmed milk in TBS-T (STAT1/3, Erk1/2) or 5% BSA in TBS-T (pSTAT1/3, pErk1/2, myc) at 4 °C overnight. After washing, the membranes were incubated with secondary peroxidase-conjugated antibodies diluted in 5% fat-free dried skimmed milk in TBS-T for 1 h at room temperature. The ECL Prime Western Blotting Detection Reagent (GE Healthcare, Freiburg, Germany) and the ChemoCam Imager (INTAS Science Imaging Instruments GmbH, Göttingen, Germany) were used for signal detection. For re-probing with another primary antibody, the membranes were stripped in 62.5 mM Tris-HCl pH 6.8, 2% SDS and 0.1% β-mercaptoethanol for 30 min at 60 °C and blocked again.

### Cell surface detection of cytokine receptors

To detect cell surface expression of the (chimeric) cytokine receptors, stably transduced Ba/F3-gp130 cell lines were washed with FACS buffer (PBS containing 1% BSA) and incubated at 5 × 10^5^ cells/100 µl FACS buffer supplemented with mIL-23R, mIL-12Rβ1 and mIL-12Rβ2 mAbs (R&D Systems) for 2 h on ice. After a single wash with FACS buffer, cells were incubated in 100 µl FACS buffer containing Alexa Fluor 647 conjugated Fab goat anti-rat IgG (Dianova) or PE mouse Armenian and Syrian hamster IgG cocktail (BD Biosciences) for 1 h at 4 °C. Finally, cells were washed once with FACS buffer, suspended in 500 µl FACS buffer and analyzed by flow cytometry (BD FACSCanto II flow cytometer, BD Biosciences). Data was evaluated using the FCS Express software (De Novo Software, Los Angeles, CA, USA).

## Electronic supplementary material

Supplementary information
