# Supplementary material for: IL-6/IL-12 Cytokine Receptor Shuffling of Extra- and Intracellular Domains Reveals Canonical STAT Activation via Synthetic IL-35 and IL-39 Signaling
Source: Sci Rep. 2017 Nov 9;7:15172. doi: 10.1038/s41598-017-15173-3 (PMC5680241; doi:10.1038/s41598-017-15173-3)

IL-6/IL-12 CYTOKINE RECEPTOR SHUFFLING OF EXTRA- AND INTRACELLULAR DOMAINS REVEALS CANONICAL STAT ACTIVATION VIA SYNTHETIC IL-35 AND IL-39 SIGNALING

D.M. Floss, M. Schönberg, M. Franke, F.C. Horstmeier, E. Engelowski, A. Schneider, E.M. Rosenfeldt & J. Scheller

Institute of Biochemistry and Molecular Biology II, Medical Faculty, Heinrich-Heine-University, 40225 Düsseldorf, Germany. [§]Correspondence should be addressed to JS (email: jscheller@uni-duesseldorf.de) and DMF (Doreen.Floss@uni-duesseldorf.de)

Supplemental Figure Legends

Figure S1. Cellular proliferation of Ba/F3-gp130 and Ba/F3-gp130/IL-12Rβ2 cells. Parental Ba/F3 cells expressing WSX-1 have been genetically modified with cDNAs for human gp130 and murine IL-12Rβ1. Equal numbers of cells were cultured for 3 days in the presence of HIL-6, recombinant murine IL-27 and recombinant human IL-35-Fc as indicated. Proliferation was measured using the colorimetric CellTiter-Blue Cell Viability Assay. HIL-6–induced proliferation was set to 100%. One representative experiment out of two is shown. Error bars represent SD. Statistical analysis used a Welch t test ($n = 3$; *$p \leq 0.05$; **$p \leq 0.01$; ***$p \leq 0.001$).

SFigure 2. (**A**) Representative histograms of all (chimeric) receptor cell surface expression in single transduced Ba/F3-gp130 cell lines (light solid lines) tested in this study. Gray-shaded areas indicate Ba/F3-gp130 cells (negative control). (**B**) Western blotting of all (chimeric) receptors in transiently transfected COS-7 cells. pEGFP-transduced cells served as negative control. The blot has been assembled from three independent Western blots.

SFigure 3. Analysis of IL-12 and IL-23 induced signal transduction. (**A**) Analysis of STAT1/3 and Erk1/2 activation. Ba/F3-gp130/IL-12Rβ1, Ba/F3-gp130/IL-12Rβ2 and Ba/F3-gp130/IL-12Rβ1/IL-12Rβ2 cells were washed three times, starved, and stimulated with 10 ng/ml HIL-6 or 4 ng/ml HIL-12 for 30 min. Cellular lysates were prepared, and equal amounts of total protein (50 μg/lane) were loaded on SDS gels, followed by immunoblotting using specific antibodies for phospho-STAT1/3/Erk1/2 and STAT1/3/Erk1/2. Western blot data show one representative experiment out of three. (**B**) Cellular proliferation of Ba/F3-gp130/IL-12Rβ1, Ba/F3-gp130/IL-12Rβ2 and Ba/F3-gp130/IL-12Rβ1/IL-12Rβ2 cells. Equal numbers of cells were cultured for 3 days in the presence of 4 ng/ml HIL-12. Proliferation was measured using the colorimetric CellTiter-Blue Cell Viability Assay. HIL-6–induced proliferation (10 ng/ml) was set to 100%. One representative experiment out of three is shown. Error bars represent SD. Statistical analysis used a Welch t test ($n = 3$; *$p \leq 0.05$; **$p \leq 0.01$; ***$p \leq 0.001$). (**C**) Analysis of STAT1/3 and Erk1/2 activation. Ba/F3-gp130/IL-12Rβ1, Ba/F3-gp130/IL-23R and Ba/F3-gp130/IL-12Rβ1/IL-23R cells were washed three times, starved, and stimulated with 10 ng/ml HIL-23 for 30 min. Cellular lysates were prepared, and equal amounts of total protein (50 μg/lane) were loaded on SDS gels, followed by immunoblotting using specific antibodies for phospho-STAT1/3/Erk1/2 and STAT1/3/Erk1/2. Western blot data show one representative experiment out of three. (**D**) Cellular proliferation of Ba/F3-gp130/IL-12Rβ1, Ba/F3-gp130/IL-23R and Ba/F3-gp130/IL-12Rβ1/IL-23R. Equal numbers of cells were cultured for 3 days in the presence of 10 ng/ml HIL-23. Proliferation was measured using the colorimetric CellTiter-Blue Cell Viability Assay. HIL-6–induced proliferation (10 ng/ml) was set to 100%. One representative experiment out of three is shown. Error bars represent SD. Statistical analysis used a Welch t test ($n = 3$; ns = not significant; ***$p \leq 0.001$). **(E)** Analysis of STAT3 target gene expression of Pim-1 in Ba/F3-gp130 cells stably transduced with IL-12Rβ1 and IL-12Rβ2 stimulated with 4 ng/ml IL-12 for 2 h. One representative experiment out of two is shown. (**F**) Western blotting of secreted HIL-23 and HIL-12 from CHO-K1 cells.

Figure S1

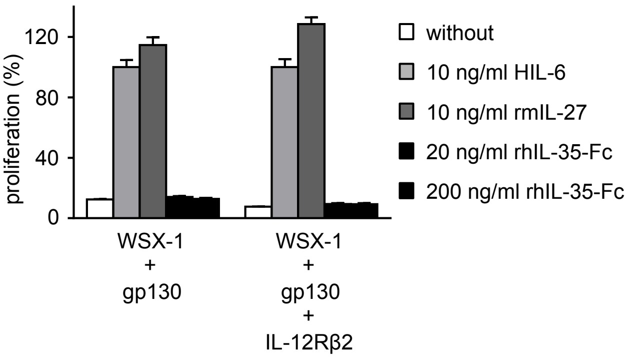

120
80
40
0
proliferation (%)
WSX-1
+
gp130
WSX-1
+
gp130
+
IL-12Rβ2
without
10 ng/ml HIL-6
10 ng/ml rmIL-27
20 ng/ml rhIL-35-Fc
200 ng/ml rhIL-35-Fc

Figure S2

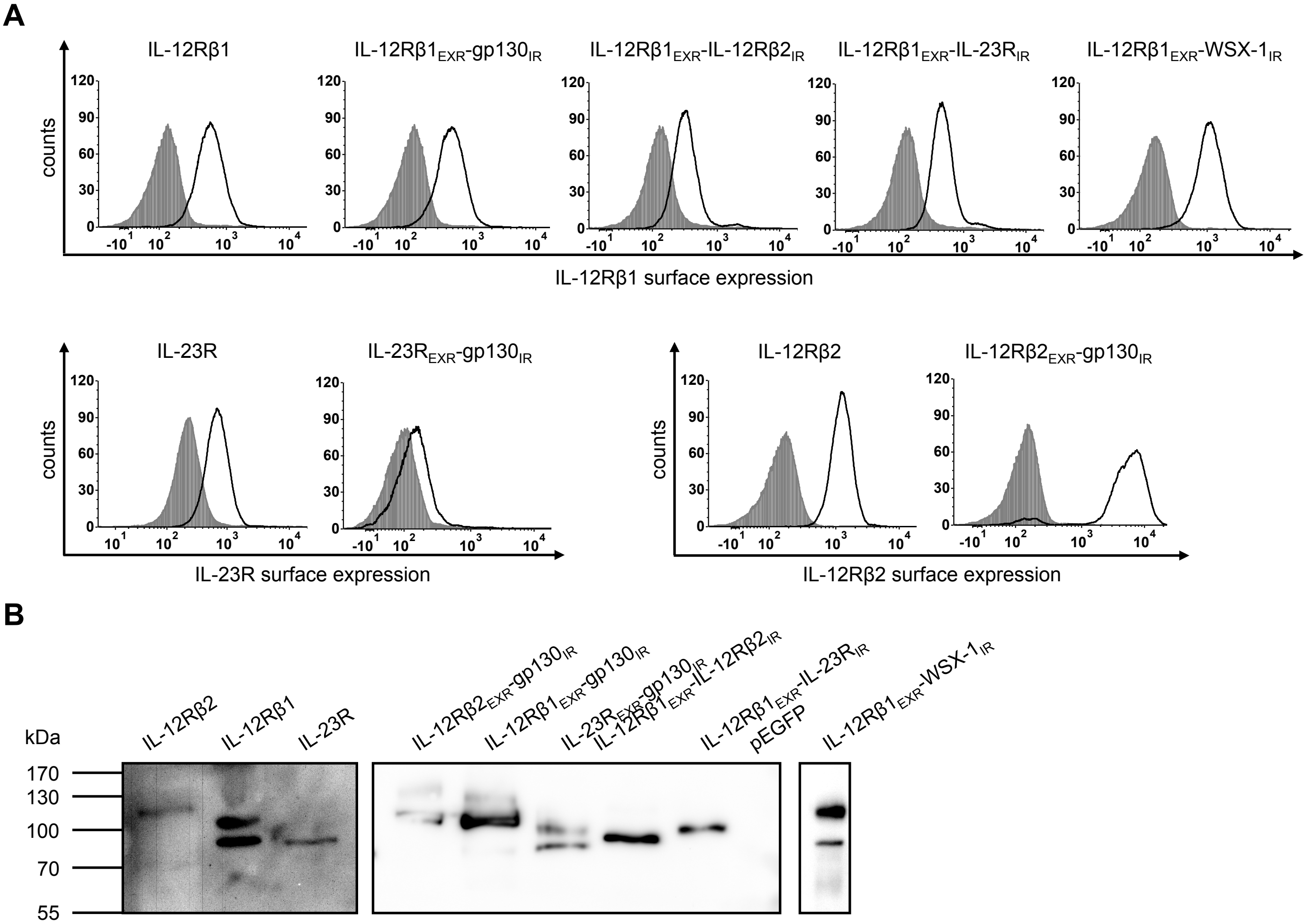
A
IL-12Rβ1
IL-12Rβ1EXR-gp130IR
IL-12Rβ1EXR-IL-12Rβ2IR
IL-12Rβ1EXR-IL-23RIR
IL-12Rβ1EXR-WSX-1IR
counts
IL-12Rβ1 surface expression
IL-23R
IL-23REXR-gp130IR
IL-23R surface expression
IL-12Rβ2
IL-12Rβ2EXR-gp130IR
IL-12Rβ2 surface expression
B
IL-12Rβ2
IL-12Rβ1
IL-23R
IL-12Rβ2EXR-gp130IR
IL-12Rβ1EXR-gp130IR
IL-23REXR-gp130IR
IL-12Rβ1EXR-IL-12Rβ2IR
IL-12Rβ1EXR-IL-23RIR
pEGFP
IL-12Rβ1EXR-WSX-1IR
kDa
170
130
100
70
55

Figure S3

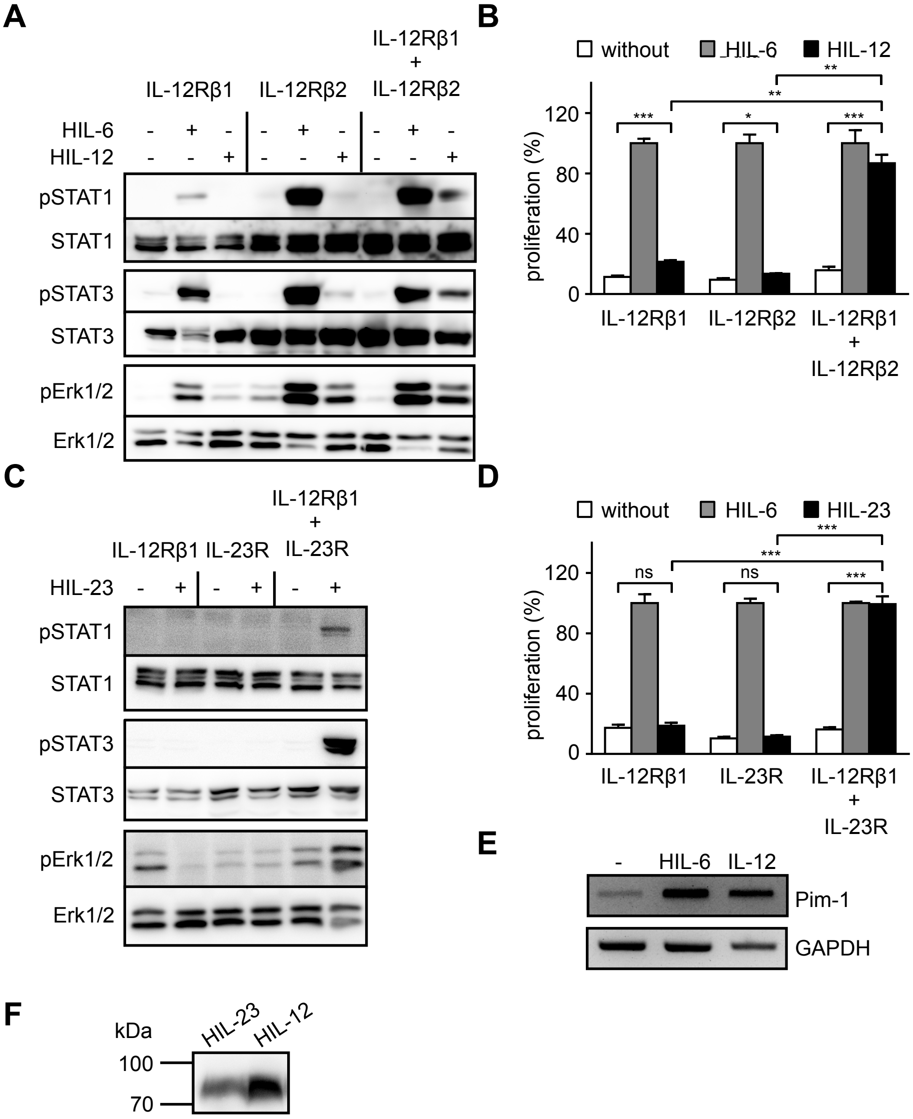

A
IL-12Rβ1
IL-12Rβ2
IL-12Rβ1 + IL-12Rβ2
HIL-6
HIL-12
pSTAT1
STAT1
pSTAT3
STAT3
pErk1/2
Erk1/2
B
without
HIL-6
HIL-12
proliferation (%)
120
80
40
0
IL-12Rβ1
IL-12Rβ2
IL-12Rβ1 + IL-12Rβ2
C
IL-12Rβ1
IL-23R
IL-12Rβ1 + IL-23R
HIL-23
pSTAT1
STAT1
pSTAT3
STAT3
pErk1/2
Erk1/2
D
without
HIL-6
HIL-23
ns
proliferation (%)
IL-12Rβ1
IL-23R
IL-12Rβ1 + IL-23R
E
-
HIL-6
IL-12
Pim-1
GAPDH
F
kDa
HIL-23
HIL-12
100
70

Assembled Western blot data for figures 2M and 6C

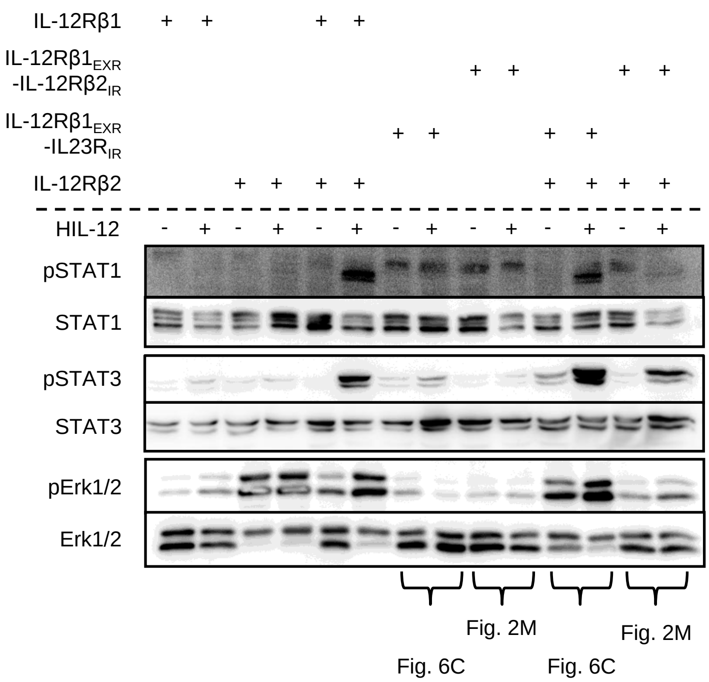

Assembled Western blot data for supplemental figure S2B

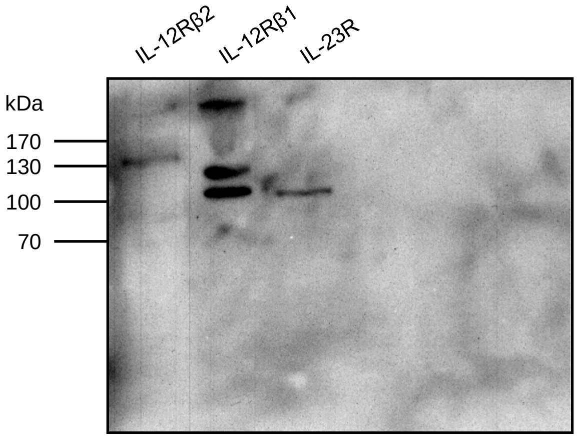

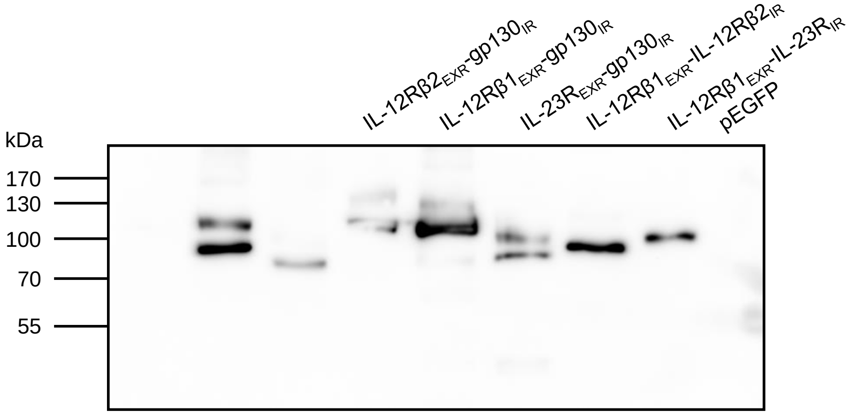

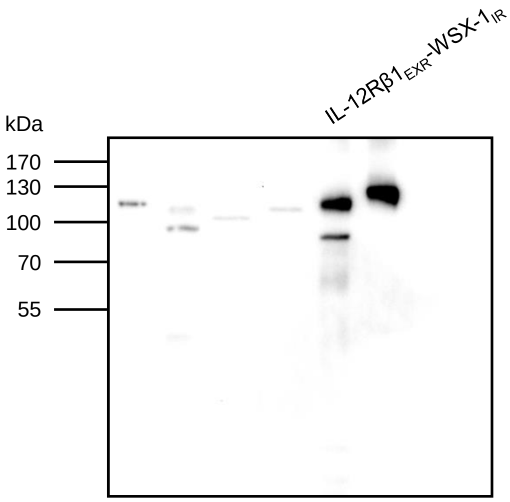

Supplement: Supplementary file 1 — Supplementary information [file 41598_2017_15173_MOESM1_ESM.pdf]
